# Optimization of offshore wind farm inspection paths based on K-means-GA

**DOI:** 10.1371/journal.pone.0303533

**Published:** 2024-05-23

**Authors:** Zhongbo Peng, Shijie Sun, Liang Tong, Qiang Fan, Lumeng Wang, Dan Liu

**Affiliations:** School of Shipping and Naval Architecture, Chongqing Jiaotong University, Chongqing, China; Università di Pisa, ITALY

## Abstract

As global demand for offshore wind energy continues to rise, the imperative to enhance the profitability of wind power projects and reduce their operational costs becomes increasingly urgent. This study proposes an innovative approach to optimize the inspection routes of offshore wind farms, which integrates the K-means clustering algorithm and genetic algorithm (GA). In this paper, the inspection route planning problem is formulated as a multiple traveling salesman problem (mTSP), and the advantages of the K-means clustering algorithm in distance similarity are utilized to effectively group the positions of wind turbines, thereby optimizing the inspection schedule for vessels. Subsequently, by harnessing the powerful optimization capability and robustness of genetic algorithms, further refinement is conducted to search for the optimal inspection routes, aiming to achieve cost reduction objectives. The results of simulation experiments demonstrate the effectiveness of this integrated approach. Compared to traditional genetic algorithms, the inspection route length has been significantly reduced, from 93 kilometers to 79.36 kilometers. Simultaneously, operational costs have also experienced a notable decrease, dropping from 141,500 Chinese Yuan to 125,600 Chinese Yuan.

## 1 Introduction

In recent years, with the increasing global demand for clean energy, the wind power industry has been thriving [1]. Compared to onshore wind farms, offshore wind farms have higher electricity generation potential due to their location in high wind speed and low turbulence environments at sea. However, due to the complex infrastructure and large grid spans of offshore wind farms [2], coupled with high installation and maintenance costs [3,4], the electricity generation costs of offshore wind power remain relatively high. According to a report by the Renewable Energy Consultation Committee (2010), operational and maintenance costs represent a significant proportion of the entire lifecycle costs of offshore wind farms, accounting for approximately 20%-35% of the total costs [5]. Consequently, recent research has increasingly emphasized the operational aspects of offshore wind farms, with a focus on optimizing operations and maintenance to reduce overall costs [6,7].

In maintenance routing and scheduling for offshore wind farms, the primary objective is to obtain detailed maintenance routes for each vessel in order to minimize the total Operation and Maintenance (O&M) costs [8]. In past research, routing and scheduling problems have consistently been a hot topic within the operations research field, with maintenance routing and scheduling for offshore wind farms garnering increasing attention as a complex optimization problem.

In recent years, routing and scheduling problems, particularly the Traveling Salesman Problem (TSP) and its various extensions and variants, have remained a highly focused research direction within the field of operations research. Researchers have employed various heuristic algorithms to address the Multiple Traveling Salesman Problem (MTSP) and seek efficient solutions. Thanya Ramanathan et al. [9] applied a genetic algorithm to solve the closed-path multiple-depot MTSP problem. They combined elite selection operators, 2-opt mutation, and Order crossover (OX crossover) methods to further optimize solutions under the guidance of reinforcement learning. Meanwhile, Ilyass Mzili et al. [10] introduced the Discrete Penguin Search Optimization Algorithm (PeSOA), which successfully addressed the Multiple Traveling Salesman Problem (MTSP). Through experiments, they demonstrated the efficiency of PeSOA. In another study, GK Baydogmus et al. [11] utilized elite ant colony optimization techniques to address the TSP/MTSP problem. They employed the parallel K-Means-Elitist Ant Colony method and achieved significant performance improvements. Additionally, Konstantin Kloster et al. [12] proposed the Multiple Traveling Salesman Problem with Drones (mTSP-DS), optimizing delivery time and energy consumption through iterative local search metaheuristics and a set-partitioning model to determine optimal tour combinations. Although these studies have achieved success in addressing traditional MTSP problems, the complexity of offshore inspection vessel routing and scheduling necessitates consideration of factors such as the number of vessels and transportation constraints like energy consumption costs. Therefore, existing heuristic algorithms require further improvement to better meet the optimization needs of offshore inspection vessel tasks.

In the domain of offshore maintenance, although the majority of research has concentrated on path optimization problems, future studies confront new challenges due to limiting factors such as the number of vessels and energy consumption costs. In the maintenance of offshore wind farms, routing and scheduling problems are of paramount importance. Dai et al. [13] proposed effective route allocation schemes through mathematical modeling and cost optimization. Stålhane et al. [14] introduced two models that utilize arc flow and path flow formulations for solution, achieving solutions close to optimality with shorter computation times. Dawid, McMillan, and Revie [15] proposed a novel heuristic approach based on the IBM CPLEX Optimizer, capable of generating near-optimal strategies within seconds. Fan et al. [16] utilized a hybrid particle swarm algorithm and discrete wolf pack search to identify optimal vessel allocation solutions. Allal et al. [17] optimized maintenance routes and scheduling for offshore wind farms using an ant colony system algorithm. O’Neil et al. [18] jointly optimized maintenance and orientation problems using a mixed-integer linear programming model and column generation method. Silva et al. [19] minimized the total route cost for maintenance service vessels servicing floating offshore wind farms through a mixed-integer linear programming approach. Leikou et al. [20] integrated genetic algorithms and particle swarm optimization to formulate patrol paths for offshore wind farms, validating the efficacy of both algorithms through simulation. Eduardo J et al [21] combines genetic algorithm (GA) and integer linear programming (ILP) to propose a new method for optimizing the layout of multi-substation wind farms, aiming to minimize infrastructure costs and power loss. Lavinia Amorosi et al [22] uses mixed integer linear programming (MILP) models to provide a solution that minimizes installation costs and weather uncertainties to address optimization challenges during offshore wind farm installation planning. Chandra Ade Irawan et al [23] addresses the logistical challenge of optimizing maintenance activities for selected turbines in offshore wind farms by coordinating a service operation vessel (SOV) and a safe transfer boat (STB), utilizing a mixed-integer linear programming model to minimize total maintenance costs. The aforementioned studies have largely achieved significant results in optimizing vessel inspection routes and scheduling, utilizing many efficient methods for similar Nondeterministic Polynomial (NP) class problems. However, there is still room for improvement in research methods for multi-constraint problems..

In summary, existing research often overlooks the constraints of practical transportation resources, such as the number of vessels and energy costs, in maintenance strategies for offshore wind farms. This paper addresses multi-constraint problems by proposing an approach that combines K-means clustering and Genetic Algorithm (GA) for the comprehensive optimization of inspection and maintenance routes and schedules in offshore wind farms.

The structure of the paper is as follows: Section 2 introduces the theoretical foundation of the Multiple Traveling Salesman Problem (MTSP) and constructs a scheduling and path optimization model for maintenance vessels in offshore wind farms. Section 3 provides detailed requirements for data on turbines, vessels, and personnel, explaining how to utilize K-means clustering and Genetic Algorithm to optimize the scheduling, inspection paths, and costs of vessels. Section 4 compares traditional Genetic Algorithm with the K-means-GA algorithm proposed in this paper through simulation experiments, demonstrating its significant advantages in reducing inspection distance and lowering operational costs. Section 5 summarizes the research findings, validating the effectiveness of the new approach.

The main contributions of this paper are as follows:

1. By considering the constraint of the number of vessels, this study transforms the inspection path problem of offshore wind farms into a Multiple Traveling Salesman Problem, thereby constructing a modeling framework for the inspection paths in offshore wind farms.
2. Integrating the goal of reducing energy consumption costs, the study optimizes the scheduling of inspection vessels using the advantages of K-means clustering in distance similarity. Subsequently, a Genetic Algorithm (GA) is employed to further refine the inspection routes, achieving effective cost reduction.
3. Compared to traditional genetic algorithms, the proposed K-means-GA hybrid method demonstrates significant advantages in reducing sailing distances and lowering inspection costs. Simulation results validate its ability to decrease inspection distance (from 93 km to 79.36 km) and operational costs (from 141,500 Chinese Yuan to 125,600 Chinese Yuan).

## 2 Modeling and description of offshore wind turbine inspection paths based on the Multi-Traveling Salesman Problem (MTSP)

In offshore wind farm inspections, properly planning and optimizing the routes of inspection vessels are crucial for enhancing inspection efficiency. This study aims to address the problem of offshore wind farm inspection routes by modeling it as a Multiple Traveling Salesman Problem (MTSP).

### 2.1 Description of the MTSP problem

The well-known Traveling Salesman Problem (TSP) involves optimizing the route of a single salesman who must visit each city exactly once. On the other hand, the Multiple Traveling Salesman Problem (MTSP) is a derivative of the TSP, where the objective is to determine the fastest paths for a group of salesmen to travel between a specified number of destinations [24]. Due to the necessity of coordinating multiple salesmen to effectively visit all cities, the problem becomes more complex in MTSP.

The optimal sequencing problem in inspection path planning is analogous to the MTSP. Let *N* be the total number of cities (including the central city), where *N* = *n* + 1 with *n* being the number of cities to be visited, and 1 representing the central city.

*d*_*ij*_:The distance (or cost) between city *i* and city *j*, where *i*, *j* = 1, 2, …, *N*.

*x*_*ijk*_:Each subscript *k* represents a traveling salesman traveling directly from city *i* to city *j* where *k* takes values from 1 to *K*, with *K* being the total number of traveling salesmen.

*u*_*i*_:The visit order of city *i* is represented by *u*_*i*_, an integer indicating the position of city *i* in the path. Typically, these integers range from 2, 3…, *N*, where 1 represents the central city.

The objective function can be expressed as:

$$Min\sum_{\left(k-1\right)}^{K}\sum_{\left(i-1\right)}^{N}\sum_{\left(j-1\right)}^{N}d_{ij}\cdot x_{kij}$$ (1)

This objective function represents the total distance or total cost for all traveling salesmen. Additionally, the MTSP is subject to the following constraint restrictions:

1. Each city can be visited by only one traveling salesman and only visited once
$$\sum_{K=1}^{K}x_{kij}=1,\forall i,j=2,3,\ldots ,N$$ (2)
2. Each traveling salesman must depart from and return to the central city.
$$\sum_{\text{j}=2}^{\text{N}}x_{k1j}=1,\forall \text{k}=1,2,3,\ldots ,\text{K}$$ (3)
$$\sum_{\text{i}=2}^{\text{N}}x_{ki1}=1,\forall \text{k}=1,2,3,\ldots ,\text{K}$$ (4)
3. The path of each traveling salesman must be continuous.
$$u_{i}-u_{j}+1\leq (N-1)\cdot {\text{(1-x}}_{kij}\text{),}\forall \text{i,j}=1,2,3,\ldots ,\text{N,}\forall \text{k}=1,2,3,\ldots ,\text{K}$$ (5)

These constraint conditions ensure the logical consistency of the problem, where the third constraint ensures that the paths of the salesmen are contiguous, avoiding the occurrence of subpaths.

### 2.2 Offshore wind farm inspection path modeling

**Fig 1:**
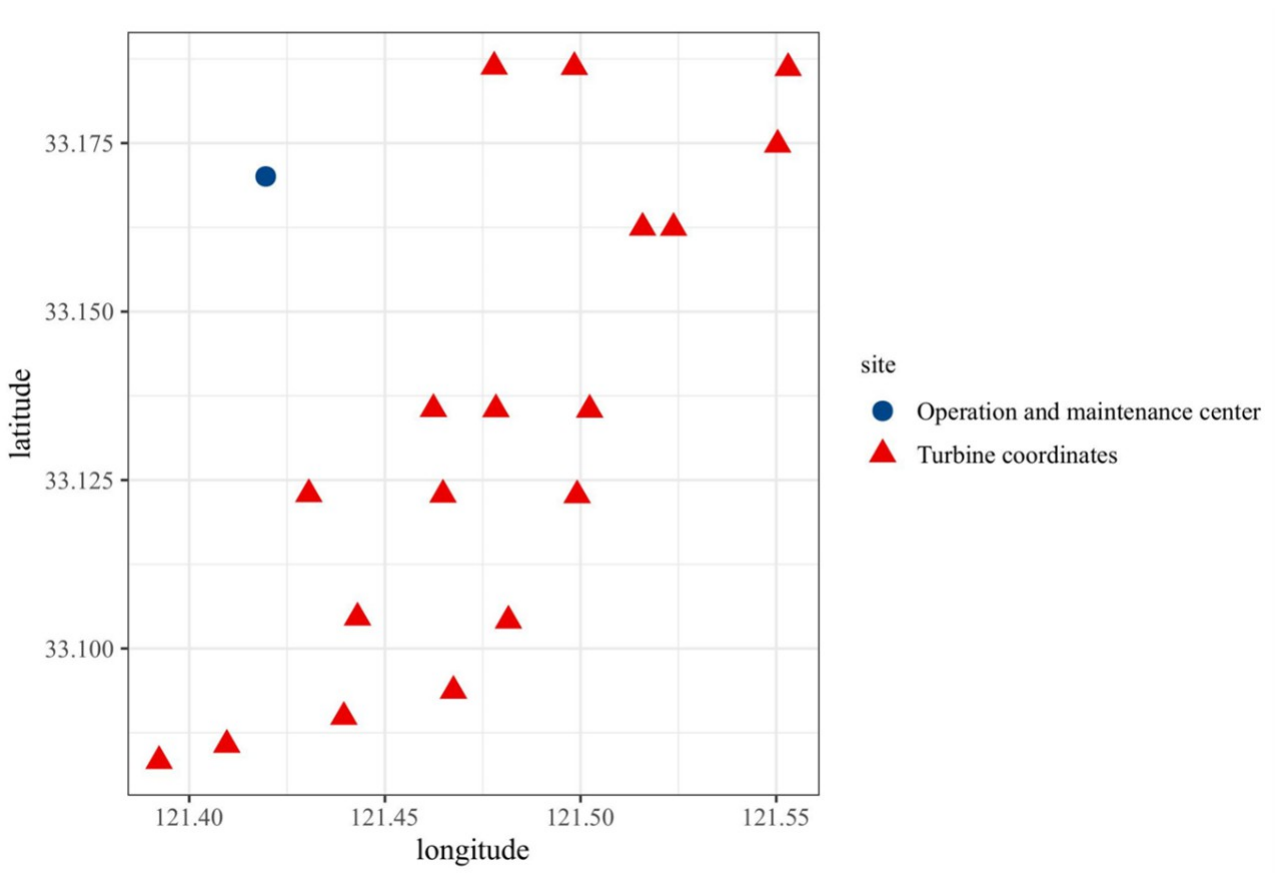
Coordinates of 18 turbines requiring maintenance in the Yellow Sea offshore wind farm.

An offshore wind farm typically consists of hundreds of turbines, but not all turbines require maintenance at the same time. Therefore, it is not necessary to visit all turbines on every occasion. The inspection route of a wind farm can be described as an MTSP problem. Fig 1 illustrates the distribution of 18 turbines that require maintenance in a particular offshore wind farm.

The problem description of the optimal access sequence in patrol route planning for offshore wind farms is as follows: Taking the coordinates of the inspection center as the starting city, the target turbine groups awaiting maintenance are considered as other cities. The straight-line distance between turbine groups can be defined as the shortest distance between two turbine groups.

Assuming there are n target turbines *W* = {*W*_1_, *W*_2_, …, *W*_*n*_}, The objective is to find the shortest path passing through all target turbines in the set W. And *D* = [*d*_*ij*_]_n*n_ (*i*, *j* = 1, 2, 3, …, *n*) is set as the distance matrix between *w*_*i*_ and *w*_*j*_. Since *W* consists of *n* turbines, there are *n*! combinations of patrol paths.

However, all closed paths share the same starting point, resulting in (*n* − 1)/2 closed paths. A closed path *x* can be represented as

$$x=\left\{W_{a(1)},W_{a(2)},\ldots ,W_{a(n)}\right\}$$ (6)

The inspection path in the middle traverses from *W*_*a*(1)_, then passes through *W*_*a*(2)_, *W*_*a*(3)_, …, *W*_*a*(*n*−1)_, and reaches the endpoint *W*_*a*(*n*)_. The solution set for the problem can be represented as:

$$S=\left\{x:\{a(1),a(2),\ldots ,a(n)\}\right\}$$ (7)

The path function *f*(*x*) representing the total length of the path is defined as:

$$f(x)=\sum_{k=1}^{n-1}d_{a(k),a(k+1)}\text{+}d_{a(n),a(1)}$$ (8)

Here, *a*(*k*) represents the *k*-th node in path a. The last term *d*_*a*(*n*),*a*(1)_ is used for closed paths, connecting the last node of the path to the first one. Therefore, the problem of finding the shortest inspection path can be formulated as:

$$f(x*)=\underset{x\in S}{\text{min}}\{f(x)\}$$ (9)

Through the above analysis, the optimal inspection path planning for offshore wind turbine arrays becomes the problem of finding the minimum value *f*(x*).

## 3 Optimization of inspection path and scheduling for offshore wind farms

Due to the complexity and flexibility of the patrol route planning and scheduling problem in offshore wind farms, the time required to find the optimal solution using exact algorithms will increase exponentially. Moreover, the optimization problem of patrol routes in offshore wind farms, similar to the MTSP problem, belongs to the NP (Nondeterministic Polynomial) class of problems, and there are currently no exact algorithms suitable for large-scale MTSP. Therefore, to address this issue, this paper proposes an optimization method based on the K-means-GA algorithm. This method aims to optimize ship scheduling, patrol route design, and find the shortest path and optimal cost.

**Fig 2:**
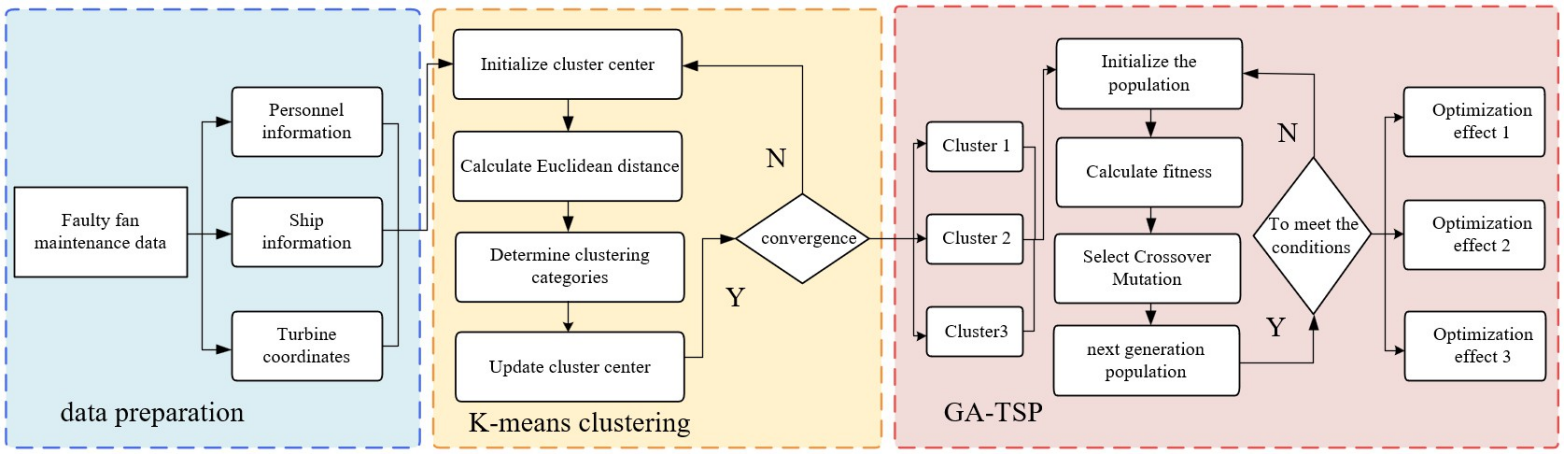
illustrates the inspection path and cost optimization design process for offshore wind farms.

Specifically, the optimization design process is illustrated in Fig 2. Firstly, by reading the coordinates of the turbines to be maintained and relevant information about maintenance vessels and personnel, the K-means clustering algorithm is employed to group the turbines based on the number of inspection vessels, facilitating the scheduling of maintenance tasks for vessels. Subsequently, the genetic algorithm (GA) is employed to conduct effective global search optimization of the inspection paths and inspection costs for each vessel through selection, crossover, and mutation, aiming to achieve the search and approximation of the global optimal solution.

### 3.1 Data preparation

In Fig 1, there are 18 wind turbine groups that need maintenance, and there is an inspection and scheduling center as the starting point for the maintenance vessel. The coordinates of the target turbine groups and the starting point are listed in Table 1. The coordinates are numbered, with 0 representing the departure point, and the rest representing the turbine coordinates. The corresponding inspection vessel information and personnel information are shown in Tables 2 and 3, respectively. In Table 2, the leasing cost of the vessels represents the cost required for each vessel to perform a complete inspection operation. In Table 3, the personnel working cost represents the wages required for each technical personnel to perform a complete inspection operation, depending on their respective categories.

**Table 1:** Coordinates of target wind turbines.

Index	latitude	longitude	Index	latitude	longitude
0	033°10′12.210″	121°25′10.338″	10	033°08′07.975″	121°27′44.655″
1	033°06′16.530″	121°26′34.798″	11	033°08′07.835″	121°28′42.127″
2	033°04′59.930″	121°23′32.239″	12	033°08′07.611″	121°30′08.335″
3	033°05′08.574″	121°24′34.561″	13	033°07′22.474″	121°25′50.016″
4	033°11′10.869″	121°28′40.518″	14	033°07′22.196″	121°27′53.430″
5	033°11′10.677″	121°29′54.271″	15	033°07′21.884″	121°29′56.842″
6	033°11′10.108″	121°33′10.943″	16	033°06′14.901″	121°28′53.618″
7	033°09′44.751″	121°31′25.561″	17	033°05′23.488″	121°26′22.242″
8	033°09′44.832″	121°30′57.065″	18	033°05′37.425″	121°28′03.055″
9	033°10′29.190″	121°33′01.298″			

**Table 2:** Ship information.

Index	Rental fee (ten thousand CNY)	Speed (km/h)	Fuel consumption (10,000 CNY /km)
1	0.5	18.5	0.11
2	0.5	17	0.12
3	0.5	16	0.09

**Table 3:** Personnel information.

Personnel category	Index	Working cost (ten thousand CNY)
technician A	1000	0.5
technician B	1001	0.6
technician C	1002	0.7

### 3.2 Optimization of patrol ship scheduling based on K-means clustering

In the research on optimizing inspection paths in offshore wind farms, this study employs the K-means clustering method, leveraging its advantages in similarity computation, local search efficiency, and rapid convergence. Addressing the complexity of the Multiple Traveling Salesman Problem (MTSP), this algorithm effectively reduces problem dimensions, simplifies mathematical models, and overcomes the liitations of traditional methods in handling high-dimensional and nonlinear problems.

In this study, the number of offshore turbines is set to n, and the number of clusters formed equals the number of maintenance vessels (*K* = *V*). The primary objective of K-means is to assign n data points to *K* clusters, such that each data point belongs to the cluster with the nearest mean. To partition the turbine coordinates, K-means first initializes a set of *K* centroids, which are randomly selected. In each iteration, each turbine is assigned to a specific cluster based on the Euclidean distance to its nearest centroid. Then, the centroid positions are recalculated using a specific formula:

$$c_{j}=\frac{1}{N_{j}}\sum_{q=1}^{N_{j}}x_{q}$$ (10)

Where *n*_*j*_ is the total number of turbines in cluster *j*, *c*_*j*_ is the centroid of cluster *j*, and *x*_*q*_ is the *q*-th turbine in cluster *j*. The process of grouping turbines to the nearest centroid and recalculating the centroids will be repeated until the termination conditions of the K-means algorithm are met.

### 3.3 Optimization of inspection ship routes and costs based on genetic algorithm

Genetic algorithms, when dealing with the Multiple Traveling Salesman Problem, are capable of conducting effective global searches compared to other heuristic algorithms. They can avoid being trapped in local optima and exhibit strong adaptability and scalability. Therefore, after assigning inspection tasks to each vessel, this section employs a genetic algorithm to optimize the inspection paths for each vessel. Since the ultimate goal of optimization is to minimize costs, the total cost includes vessel costs, personnel costs, etc. Thus, the cost minimization expression can be formulated as follows:

$$minZ=Z^{tr}+Z^{tech}$$ (11)

Ship Costs:

$$Z^{tr}=Min\sum_{v\in V}^{V}\sum_{i\in N}^{N}\sum_{j\in N}^{N}(c_{vij}\cdot x_{vij})$$ (12)

Personnel cost:

$$Z^{tech}=\sum_{p\in P}^{P}t_{ip}$$ (13)

Here, *c*_*vij*_ in the ship cost represents the travel cost of ship *v* from turbine *i* to turbine *j*. *X*_*vij*_ = 1 if and only if ship v travels from turbine *i* to turbine *j*, otherwise, it is 0. In this model, this study consider the inspection center during the outbound leg as turbine 0, and the inspection center during the return leg as turbine *N* + 1.In the personnel cost, *t*_*ip*_ denotes the requirement of *p*-th type worker for turbine *i* and *u*_*i*_ represents the visit order of turbine *i*. *u*_*i*_ is an integer representing the position of turbine *i* in the path. *V* is the set of all ships, *N* is the set of all turbines to be maintained, and *P* is the set of worker types.

Constraint conditions: The constraints of the Multiple Traveling Salesman Problem are defined according to Eqs (2) to (5).

**Fig 3:**
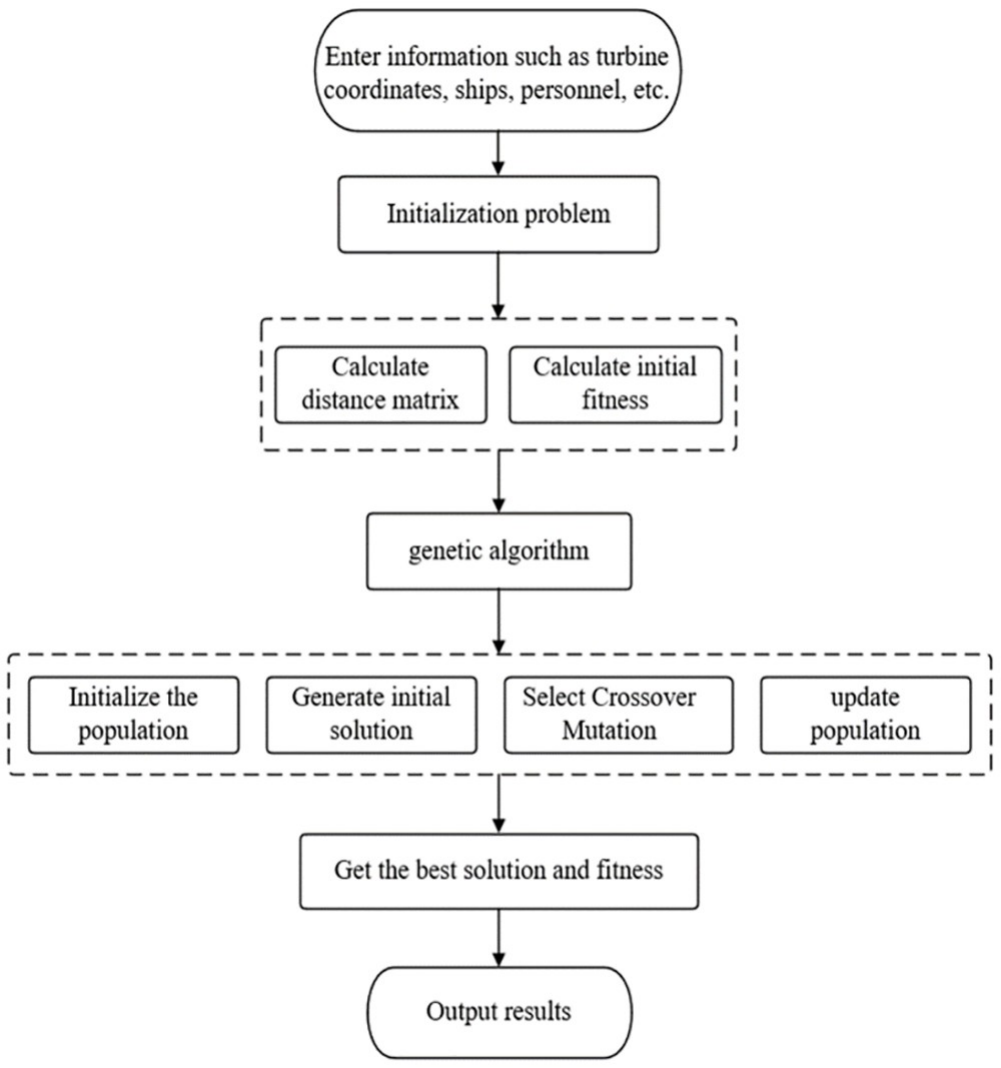
Processing flow of GA algorithm.

Based on the minimization cost model in Eq (11) and the minimization path model in Eq (9), this study employs a genetic algorithm (GA) to first solve Eq (9), thereby obtaining the optimal solution for Eq (11). By combining selection, crossover, and mutation techniques within the genetic algorithm framework, this study achieves a solution that minimizes both travel distance and inspection costs. Furthermore, compared to other evolutionary methods for solving the MTSP problem, this study groups wind turbine coordinates, allowing each vessel to be responsible for a smaller subset. This significantly reduces the length of genetic encoding for individuals in the genetic algorithm and the size of the population [25], providing a notable advantage in computational complexity for the proposed algorithm. The computational process is illustrated in Fig 3.

Based on the provided turbine coordinates, vessel, personnel, and other information, a Traveling Salesman Problem (TSP) is formulated. This problem, along with parameters such as population size, generations, parents’ number, and mutation probability, is passed to the genetic algorithm function. The function iterates through each generation for selection, mutation, and crossover, thereby generating a new population at each iteration. Then, the final population is used to calculate fitness and obtain an optimal solution, resulting in the ultimate optimal route and minimum cost. The following is the detailed solving process:

Step 1: Initial Solution Generation

Due to its characteristics of rapid generation, feasibility guarantee, local optimization, and avoidance of randomness, the greedy algorithm is chosen to generate *N* initial feasible solutions, forming the population (Pop). Each individual in the population is evaluated using Eq (9), where *t* = 1.

Step 2: Tournament Selection

Tournament Selection is employed as the selection strategy, where a random subset of individuals is compared to choose a portion of solutions to enter the next generation population. This reduces the likelihood of overlooking the best solutions in the population, thereby enhancing global search capability. A new population of size *N*, denoted as New Pop, is generated.

Step 3: Sequential Crossover Operation

Through the Order Crossover (OX) operator, randomly selected segments of genes from the parent chromosomes are copied to the corresponding positions in the offspring. Subsequently, the remaining gene segments from the other parent are excluded, and they are placed into the offspring in the order they appear, generating new solutions.

Step 4: 2-opt Mutation Operation

Employing the 2-opt mutation operator, two edges are selected in the solution, and the order of nodes between these two edges is reversed. By modifying the path of the selected nodes, a new solution equivalent to the original one is generated. New Pop is then evaluated using Eq (9).

Step 5: Update Population

The generated offspring individuals, New Pop, replace individuals in the original population Pop to facilitate the population’s evolution. Specifically, for each individual, it replaces individuals with lower fitness in the original population, ensuring that the new generation population still maintains a size of *N*. This replacement strategy helps maintain a population of excellent solutions, promoting the evolutionary direction of the population.

Step 6: Select the Top N Individuals

Choose the top N individuals to form the next generation population (Pop). If the stopping criterion is met, output Pop, and the algorithm terminates; otherwise, set *t* = *t* + 1 and proceed to Step 2.

Through the above algorithmic process, the optimal solutions for Eqs (9) and (11) can be obtained, achieving the shortest inspection path and minimal inspection cost for patrol vessels.

## 4 Experimental verification and result analysis

In this section, simulation and emulation of offshore wind farm inspection scheduling are conducted based on the inspection path and cost models proposed in Eqs (9) and (11), along with the corresponding data preparation. To validate whether the optimization effect of the algorithm proposed in this paper surpasses other algorithms, this study will compare the optimization results obtained from the K-means-GA algorithm proposed earlier with those obtained from traditional genetic algorithms.

In the algorithm used in this paper, the genetic algorithm’s population is set to 50, the maximum number of iterations is 50, the crossover probability is 0.8, and the mutation probability is 0.2.

**Fig 4:**
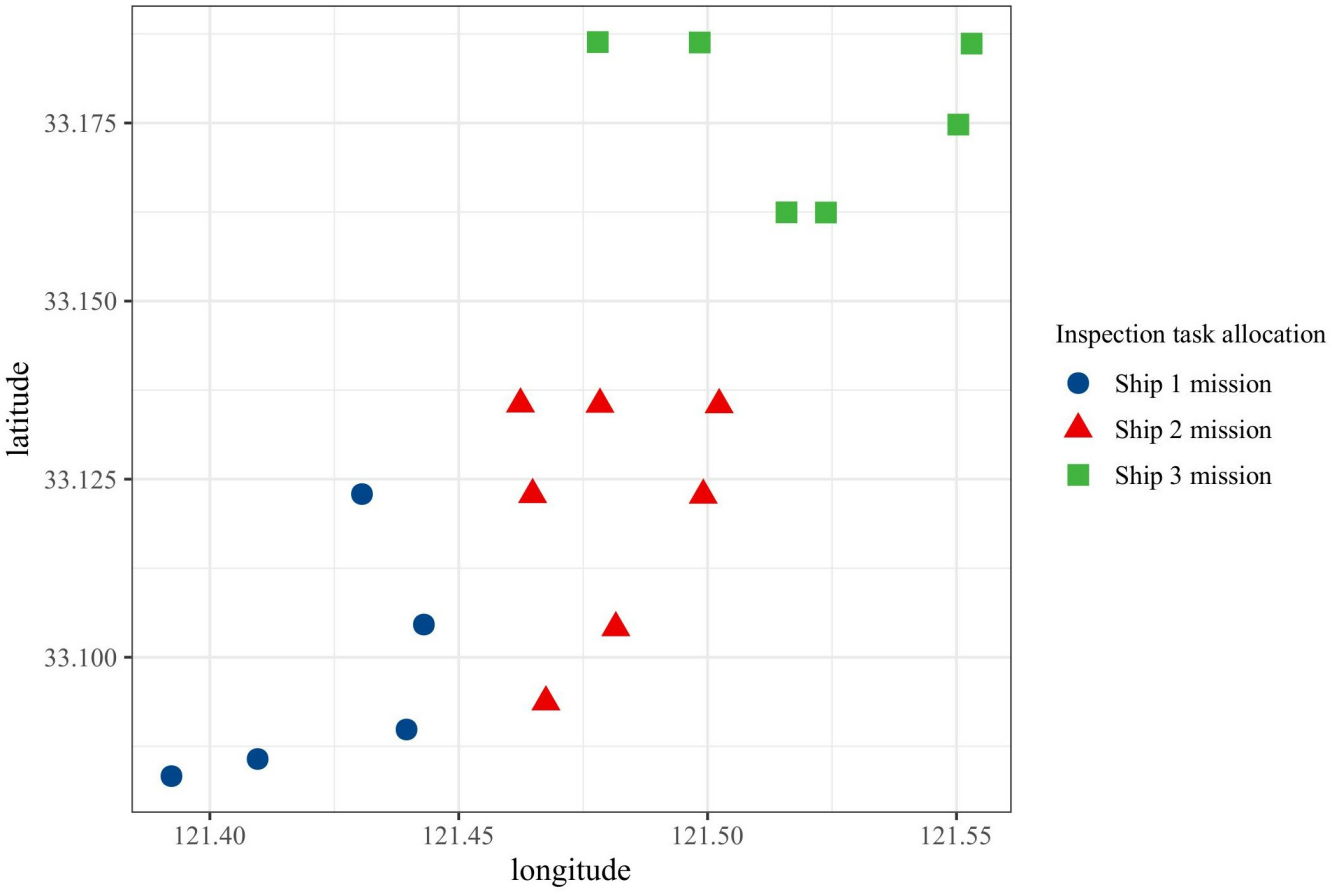
Clustering effect.

In the solving process of the K-means-GA algorithm, the turbine coordinates are first categorized into three groups based on the number of vessels specified in the vessel information, as calculated using Eq (10), as illustrated in Fig 4. Additionally, this study has allocated specific maintenance tasks to each vessel (corresponding to turbine numbers). The detailed allocation results are presented in Table 4.

**Table 4:** Scheduling tasks of ship inspection.

ship number	Wind turbine inspection task number
1	1, 2, 3, 13, 17
2	10, 11, 12, 14, 15, 16, 18
3	4, 5, 6, 7, 8, 9

**Fig 5:**
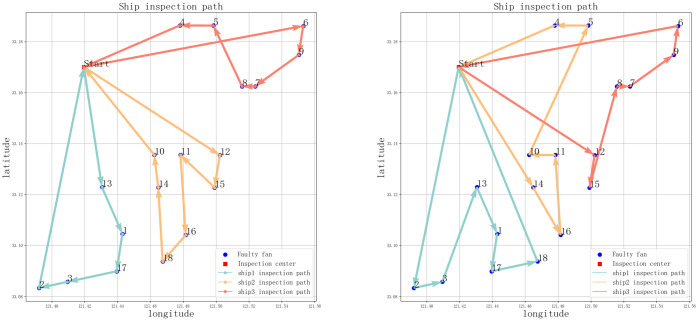
Ship polling path based on K-mean-GA algorithm and GA algorithm.

**Fig 6:**
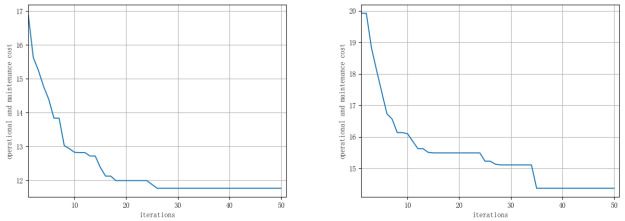
Iterative optimization process of 100 inspection costs based on K-mean-GA algorithm and GA algorithm.

After completing the ship scheduling, each vessel is assigned a set of wind turbines for maintenance. This study employs a genetic algorithm to plan the maintenance routes for each vessel. Following the process described in Section 3.3, the optimal solution for inspection paths and costs is computed. Additionally, for comparison, this paper also utilizes a traditional genetic algorithm to directly solve the Multiple Traveling Salesman Problem (MTSP). Table 5, along with Figs 5 and 6, presents the results of the two models. Specifically, Table 5 compares the optimization outcomes of ship inspection paths obtained using different algorithms. The results indicate that the K-means-GA algorithm, compared to the traditional genetic algorithm, has a significant advantage in reducing path distance and lowering inspection costs. The inspection path length decreases from 93 kilometers to 79.36 kilometers, and operational costs reduce from 141,500 Chinese Yuan to 125,600 Chinese Yuan.

**Table 5:** Results of ship inspection optimization using K-mean-GA algorithm and GA algorithm.

method	K-means-GA algorithm			GA algorithm		
ship number	Inspection path	Inspection distance (km)	Inspection cost (CNY 10000)	Inspection path	Inspection distance (km)	Inspection cost (CNY 10000)
1	13-1-17-3-2	23.77	3.85	6-9-7-8-12-15	32.39	4.89
2	12-15-11-16-18-14-10	27.87	4.34	14-10-4-5-16-18	28.49	4.41
3	4-5-8-7-9-6	28.12	4.37	11-1-17-13-3-2	32.12	4.85

Fig 5 depicts the optimization paths for ship inspection scheduling in offshore wind farms using two different algorithms. The left side shows the K-means-GA algorithm, while the right side shows the GA algorithm. It can be observed that compared to the K-means-GA algorithm, the optimal path obtained by the GA algorithm during the optimization process appears more disordered and chaotic, indicating a relatively poor quality of solution after 50 iterations. In contrast, the optimal route obtained by the K-means-GA algorithm demonstrates significant improvement compared to GA, showcasing a notably enhanced optimization outcome.

Fig 6 illustrates the iterative optimization process of inspection costs for two algorithms, with the K-means-GA algorithm on the left and the GA algorithm on the right. It can be observed that during the optimization process, between 20 and 30 iterations, the convergence speed of K-means-GA is significantly faster than that of the traditional genetic algorithm (GA). The K-means-GA algorithm achieves convergence earlier, demonstrating clear improvements over GA in both search capability and efficiency.

From the above graphs and tables, it is evident that utilizing the K-means-GA algorithm in conjunction with the MTSP problem for optimizing offshore wind farm inspection paths results in fewer inspection distances and lower inspection costs compared to traditional genetic algorithms. This indicates that the method proposed in this paper holds a clear advantage in optimizing offshore wind farm inspection paths.

## 5 Conclusion

This study aims to find the shortest paths to effectively reduce inspection costs through scheduling and path optimization based on the K-means-GA algorithm. Considering the varying degrees of aging and maintenance cycles of turbine groups, a multiple traveling salesman problem-based inspection path model for offshore wind farms is proposed. A comparison of the performance of the K-means-GA algorithm and traditional genetic algorithms in inspection path optimization reveals that the total path length optimized by the K-means-GA algorithm is 79.36 kilometers, significantly less than the 93 kilometers optimized by the GA algorithm. Moreover, the inspection cost is notably lower, amounting to 124,600 yuan compared to 141,500 yuan for the latter. Hence, the K-means-GA algorithm is more suitable for scheduling and path optimization of inspection vessels in offshore wind farms compared to the GA algorithm.

In future research, we intend to further upgrade and enhance the optimization algorithm. Our plan includes incorporating additional practical constraints such as weather conditions and inspection time to better adapt to the real-world requirements of ship inspections in offshore wind farms.
